# Identification of a Conserved Linear Antigenic Determinant in the Senecavirus A VP1 Protein

**DOI:** 10.3390/ani16121856

**Published:** 2026-06-16

**Authors:** Zhaogeng Wu, Junyao Wang, Zhe Liu, Wei Yao, Jiayi Zang, Meitong Lu, Baozhu Zhang, Dongcheng Zheng, Yu Hong, Meijun Zhou, Jiashan Sun, Xuexia Wen

**Affiliations:** 1 Key Laboratory of Livestock Infectious Diseases, Ministry of Education, and Key Laboratory of Ruminant Infectious Disease Prevention and Control (East), Ministry of Agriculture and Rural Affairs, College of Animal Science and Veterinary Medicine, Shenyang Agricultural University, 120 Dongling Road, Shenyang 110866, China; 2 Liaoning Rural and Agricultural Development Service Center, 7-1 Lingyuan Street, Shenyang 110031, China; 3 Jiangsu Lihua Foods Group Co., Ltd., 66 Caoxi Road, Changzhou 213161, China

**Keywords:** Senecavirus A, VP1, monoclonal antibody, epitope

## Abstract

Senecavirus A (SVA) is a newly emerging picornavirus associated with porcine idiopathic vesicular disease and sudden death in newborn piglets. Currently, no specific vaccines or drugs are available against SVA, highlighting the importance of investigating the immunological characteristics of its key proteins. The VP1 protein of SVA exhibits strong immunogenicity and high sequence conservation, and it is indispensable to the viral life cycle. In the present study, a monoclonal antibody (mAb) against VP1 was generated. A series of truncated VP1 proteins was then expressed to precisely map the epitope recognized by this mAb. The minimal reactive unit was identified as ^16^DTDFSGELA^24^. Homology analysis further revealed that this epitope is conserved among different SVA isolates deposited in GenBank. Moreover, AlphaFold prediction, along with PyMOL (Version 3.0.3) and GETAREA analyses, reveals that this epitope resides in the α-helix and loop regions of the three-dimensional structure of the VP1 protein and is surface-exposed. Collectively, these findings indicate that the mAb and its recognized epitope represent valuable tools for investigating SVA etiology and VP1 protein function.

## Simple Summary

Senecavirus A (SVA) is an emerging picornavirus that causes vesicular disease and neonatal death in pigs, yet no specific vaccines or drugs are available. The VP1 protein of SVA is immunogenic, highly conserved, and essential for the viral life cycle. We generated a monoclonal antibody (mAb) against VP1 and mapped its recognized epitope to the minimal unit ^16^DTDFSGELA^24^. This epitope is conserved among various SVA isolates. Structural predictions and analyses show that the epitope is located in the α-helix and loop regions of VP1 and exposed on the protein surface. Collectively, the mAb and its epitope provide valuable tools for studying SVA etiology and VP1 function.

## 1. Introduction

Senecavirus A (SVA) is a non-enveloped virus belonging to the genus *Senecavirus* within the family *Picornaviridae*, which causes vesicular disease in pigs [1,2,3,4]. Approximately 7.2 kb in length, the single-stranded positive-sense RNA genome of the virus includes a 5′ untranslated region (UTR), a 3′ UTR, a polyA tail, and an open reading frame (ORF) [3,5]. The ORF-encoded polyprotein is post-translationally cleaved, generating four structural proteins VP1–VP4 and eight non-structural proteins Lpro, 2A, 2B, 2C, 3A, 3B, 3C, and 3D [2,6].

Originally identified serendipitously in cell cultures in 2002, SVA was subsequently detected in pigs presenting with vesicular disease five years later [2,7]. However, it was not until the large-scale outbreaks of vesicular disease in pigs occurred in Brazil and the United States during 2014–2015 that SVA gained substantial attention [8,9]. In 2015, SVA infection in pigs was first reported in China, where infected newborn piglets exhibited sudden death as a novel clinical sign [10,11]. Since then, SVA infections have been documented in several countries, including Colombia, Thailand, Vietnam, Chile, and Mexico, causing considerable economic losses to the swine industry worldwide [12,13,14,15,16]. Clinically, SVA infection manifests as lethargy, anorexia, lameness, and vesicular lesions on the snout, oral mucosa, or hooves [3,17]. These clinical signs closely resemble those of other vesicular diseases, such as swine vesicular disease (SVD), vesicular stomatitis (VS), vesicular exanthema of swine (VES), and foot-and-mouth disease (FMD), making accurate clinical differentiation challenging [4,18]. Consequently, laboratory-based diagnostic methods are indispensable for definitive confirmation.

Within the Picornaviridae family, the VP1 protein is recognized as a highly immunogenic antigen [19,20]. Although its sequence similarity is low among picornaviruses from distinct genera, VP1 exhibits pronounced conservation at the species level: nucleotide sequence identity among different strains ranges from 99% to 100%, and amino acid sequence identity reaching up to 100% [21]. Accumulating evidence indicates that the VP1 protein of SVA is implicated in viral cell tropism and receptor binding [22,23,24]. Given its robust immunogenicity, high sequence conservation, and critical role in the viral life cycle, delineating the antigenic regions of VP1 is essential for elucidating SVA pathogenesis and advancing immunodiagnostic strategies.

In the present study, the VP1 protein was expressed in prokaryotes and used to immunize mice, enabling the generation of a VP1-specific monoclonal antibody (mAb) via hybridoma technology. Moreover, the linear B-cell epitope recognized by this mAb was mapped through truncated expression of VP1 fragments, thereby providing a valuable tool for virological research.

## 2. Materials and Methods

### 2.1. Viruses, Cells, Plasmids, and Main Reagents

The SVA strain CHhb17 (GenBank No. MG983756.1) was propagated and stored in our laboratory [25]. The baby hamster kidney (BHK-21) and human embryonic kidney (HEK-293T) cells were maintained in Dulbecco’s Modified Eagle’s Medium (DMEM; Thermo Fisher Scientific, Waltham, MA, USA) supplemented with 1% penicillin–streptomycin (LABGIC, Beijing, China) and 10% fetal bovine serum (FBS; NULEN biotechnology, Shanghai, China). The myeloma cells (SP2/0) and hybridoma cells were cultured in RPMI 1640 (Thermo Fisher Scientific) supplemented with 1% penicillin–streptomycin and 20% FBS. All cells were cultured under controlled conditions at 37 °C with 5% CO_2_ in a humidified incubator (Thermo Fisher Scientific). The recombinant plasmids pEGFP-N2-SVAVP1 and pCMV-Flag-SVAVP1 were previously constructed by inserting the VP1 gene sequence of SVA (CHhb17) into the pEGFP-N2 (Beijing Zoman Biotechnology Co., Ltd., Beijing, China) and p3XFLAG-CMV-10 (Sigma-Aldrich, Darmstadt, Hessen, Germany) vectors, respectively. The VP1 sequence of the FMDV type O/BY/CHA/2010 strain (GenBank No. JN998085.1) was synthesized by Beijing Ruibo Biotechnology Co., Ltd. (Beijing, China) and inserted into the pCMV-Flag vector, generating the recombinant plasmid pCMV-Flag-FMDVVP1. Ni-NTA Agarose was purchased from QIAGEN GmbH (Hilden, NRW, Germany). Freund’s adjuvant, 50 × HAT, 50 × HT, and the fusion agent PEG2000 were purchased from Sigma-Aldrich. The mouse monoclonal antibody isotyping ELISA kit was purchased from Biodragon (Suzhou, China).

### 2.2. Construction and Identification of the VP1 Prokaryotic Expression Vector

Primers were designed based on the VP1 nucleotide sequence of the CHhb17 strain and synthesized by Beijing Ruibo Biotechnology Co., Ltd. (Table 1). The VP1 gene, amplified from the SVA strain CHhb17, was inserted into the pET-28a vector via the *Bam*HI and *Hin*dIII restriction sites using a homologous recombination cloning kit (Yeasen Biotechnology, Shanghai, China). The constructed plasmids were subsequently transformed into Trans 10 competent cells and verified by DNA sequencing. After sequence confirmation, the recombinant plasmid pET-28a-VP1 was extracted.

### 2.3. Expression and Purification of Recombinant VP1 Protein

The recombinant plasmid pET-28a-VP1 was transformed into competent cells *Escherichia coli (E. coli)* BL21 (DE3) (TransGen Biotech, Beijing, China) for protein expression. A single colony was selected and inoculated into LB liquid medium supplemented with kanamycin and cultured in a shaker. Upon the OD600 reaching 0.6–0.8, IPTG was added at a final concentration of 1 mM to trigger protein expression, and the culture was incubated for an additional 6 h. The bacterial culture was centrifuged to collect the pellet, which was washed twice, resuspended in phosphate-buffered saline (PBS), and then ultrasonically disrupted on ice. Following lysis, an aliquot of the whole-cell lysate was retained for analysis. The lysate was centrifuged to separate the supernatant from the pellet. Recombinant VP1 protein expression was assessed by SDS-PAGE combined with Coomassie Brilliant Blue staining. The protein was then purified by Ni^2+^-affinity chromatography. After purification, its purity and antigenicity were evaluated by SDS-PAGE and Western blot analysis, respectively.

### 2.4. Generation of mAbs Against VP1 Protein

Six-week-old female BALB/c mice were immunized subcutaneously on the back with 20 μg per mouse of the purified recombinant VP1 protein via multi-point injection. After a two-week interval, a booster immunization was given using the same dose and route. Blood samples were collected a week following the third immunization, and serum antibody titers were measured by indirect enzyme-linked immunosorbent assay (ELISA). Splenocytes were isolated from mice with relatively high serum titers and subsequently fused with SP2/0 cells employing conventional hybridoma technology. Positive hybridomas were screened by indirect ELISA. After three consecutive cycles of subcloning and identification, a hybridoma cell line stably secreting VP1 monoclonal antibody was obtained.

### 2.5. Indirect Enzyme-Linked Immunosorbent Assay

Indirect ELISA was conducted as previously described [26,27]. Briefly, the purified VP1 proteins were coated in 96-well plates with a dose of 0.4 μg/well at 4 °C overnight. Next, 5% skimmed milk was added to block the plates. Subsequently, hybridoma supernatants were incubated on plates for 1 h at 37 °C, followed by incubation with horseradish peroxidase (HRP)-conjugated goat anti-mouse IgG (Sigma-Aldrich) for 1 h at 37 °C. Thereafter, color development was initiated by adding the substrate solution, and the reaction was terminated with 2 mol/L H_2_SO_4_. The absorbance was measured at 450 nm using a microplate reader (BioTek, Winooski, VT, USA).

### 2.6. Western Blot

After mixing with loading buffer, the cell lysates were heated at 100 °C for 5 min and separated by SDS-PAGE. Following electrophoresis, the samples were transferred electrophoretically to a nitrocellulose membrane (Epizyme Biomedical, Shanghai, China), which was then blocked with 5% skimmed milk in PBS for 1 h at 37 °C. Subsequently, the membrane was incubated overnight at 4 °C with either anti-His antibody (Proteintech Group, Wuhan, China) or hybridoma cell supernatants, followed by a 1 h incubation at 25 °C with HRP-conjugated secondary antibodies (Sigma-Aldrich). After five washes, the signal was detected using an ECL Western blot system (Tanon, Shanghai, China).

### 2.7. Indirect Immunofluorescence Assay

BHK-21 cells were either infected with the SVA strain CHhb17 at a multiplicity of infection (MOI) of 0.1 or mock-infected with RPMI 1640. At 24 h post-infection, the cells were fixed with 4% paraformaldehyde for 10 min at 25 °C and washed thrice with PBS. The immobilized SVA-infected cells were then permeabilized with PBS containing 0.1% Triton X-100 for 10 min at 25 °C, followed by blocking with 2% BSA in PBS for 30 min at 25 °C. Hybridoma cell supernatants were then added and incubated for 1 h at 25 °C. Following three washes with PBS, the cells were incubated with Alexa Fluor 488-conjugated goat anti-mouse IgG F(ab′)_2_ fragments (Thermo Fisher Scientific) for 1 h at 25 °C. Subsequently, 4′,6-diamidino-2-phenylindole (DAPI, Thermo Fisher Scientific) was used to stain nuclei for 5 min at 25 °C. Thereafter, the plates were washed thrice and examined under a fluorescence microscope (Leica Microsystems GmbH, Wetzlar, Hessen, Germany).

### 2.8. Epitope Mapping

**Figure 1:**
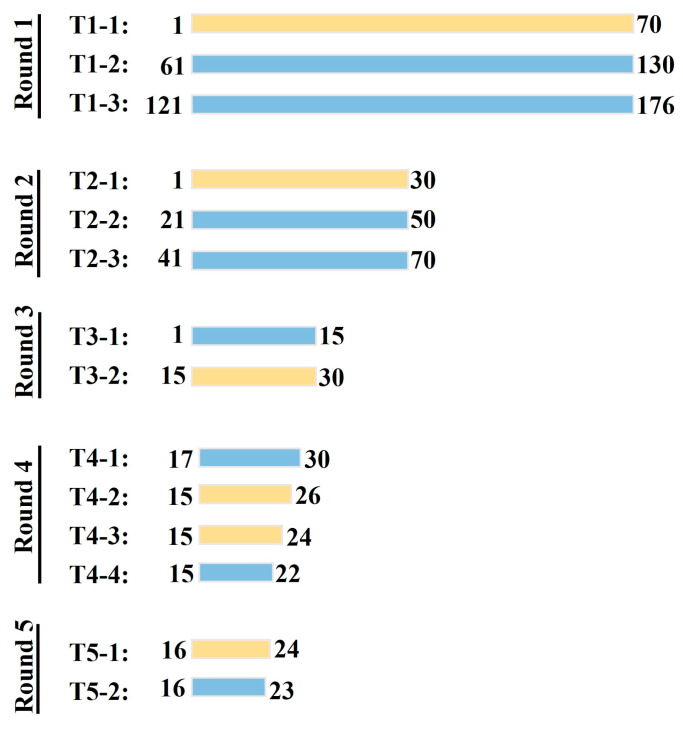
Schematic diagram showing truncated VP1 proteins used for epitope mapping. Rectangles represent truncated VP1 proteins, and numbers indicate start and end amino acid positions of different truncation variants. Light yellow rectangles denote mAb-reactive peptides, while light blue rectangles indicate non-reactive ones.

To locate the amino acid region recognized by the mAb, the VP1 protein was divided into three segments, each of which was cloned into pEGFP-N2 to generate recombinant plasmids. After transfection into BHK-21 cells, expression of the truncated proteins was confirmed by Western blot using a GFP antibody (Proteintech Group). Subsequently, the VP1 fragments recognized by the mAb were identified. A schematic representation of the VP1 peptides used for epitope mapping is shown in Figure 1. For precise localization, a series of peptides sequentially truncated from the N-terminus and C-terminus of the identified fragment were tested for reactivity with the mAb.

### 2.9. Homology Analysis

To explore the conservation of the identified epitope among SVA strains, sequence alignments were performed with the amino acid sequences of the SVA polyprotein or VP1 protein available in GenBank using the Molecular Evolutionary Genetics Analysis Version 12 (MEGA 12) software [28]. Specifically, the VP1 amino acid sequences of SVA were retrieved from the GenBank database, and multiple sequence alignment was performed using the MUSCLE algorithm in MEGA 12 with default parameters. Subsequently, the “Toggle Conserved Sites” function (with the conservation threshold set to 100%) was used to analyze the alignment and determine the conservation of this epitope.

### 2.10. Spatial Distribution Analysis of the Identified Epitope on the VP1 Protein

To analyze the spatial distribution of the identified epitope on the VP1 protein, the three-dimensional structure of the VP1 protein was predicted using AlphaFold based on its amino acid sequence [29]. The predicted structure was then imported into PyMOL software (Version 3.0.3) and the GETAREA program (https://curie.sdaponline.org/getarea.html) to visualize and analyze the spatial distribution characteristics of the epitope within the VP1 structure [30].

## 3. Results

### 3.1. Expression and Purification of Recombinant VP1 Protein

**Figure 2:**
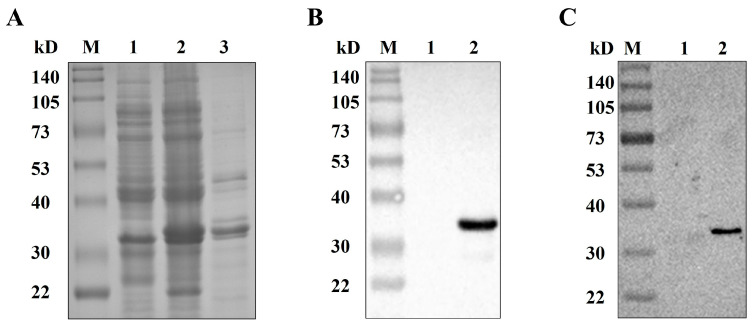
Expression and purification of the recombinant VP1 protein. (**A**) SDS-PAGE analysis of recombinant VP1 protein expression. M, protein marker; Lanes 1 and 2, IPTG- induced lysates of *Escherichia coli* (*E. coli*) transformed with pET-28a (Lane 1) or pET-28a-VP1 (Lane 2); Lane 3, purified recombinant VP1 protein. (**B**,**C**) Western blot analysis of the purified recombinant VP1 protein probed with anti-His antibody (**B**) or anti-SVA swine sera (**C**). M, protein marker; Lane 1, lysates of IPTG-induced *E. coli* harboring pET-28a; Lane 2, purified recombinant VP1 protein.

The VP1 gene was cloned into the pET-28a vector by homologous recombination, yielding the recombinant construct pET-28a-VP1. After sequence confirmation, this construct was transformed into competent cells *E. coli* BL21 (DE3), and protein expression was induced with IPTG. As shown in Figure 2A, Coomassie brilliant blue staining following SDS-PAGE revealed a prominent band between 30 and 40 kD, matching the predicted molecular mass and confirming successful expression of the recombinant VP1 protein. This protein was then purified via affinity chromatography, and SDS-PAGE analysis confirmed its high purity (Figure 2A). Western blot analysis showed that the purified VP1 protein reacted with both anti-His antibody and SVA-positive serum, indicating its good immunogenicity and suitability as an immunogen for generating anti-VP1 monoclonal antibodies (Figure 2B,C).

### 3.2. Determination of Serum Bioactivity in Mice Immunized with Recombinant VP1 Protein

**Figure 3:**
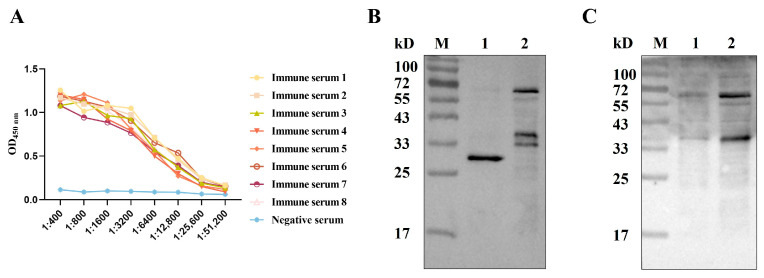
Serum bioactivity in mice immunized with recombinant VP1 protein. (**A**) Measurement of VP1-specific serum antibody titers in mice after the third booster using indirect ELISA. (**B**,**C**) Western blot analysis of the eukaryotic expressed VP1 protein with anti-GFP antibody (**B**) and VP1-immunized mouse serum (**C**). M, Marker; Lane 1 and 2, lysates from HEK-293T cells transfected with either pEGFP-N2 (Lane 1) or pEGFP-N2-SVAVP1 (Lane 2).

The purified recombinant VP1 protein was fully emulsified and used to immunize mice. A total of three immunizations were administered at two-week intervals. Sera were collected one week after the final immunization, and antibody titers were quantified via indirect ELISA. All immunized mice yielded serum antibody titers above 1:25,600 (Figure 3A). For further antigen reactivity verification, a Western blot was conducted against GFP-VP1 fusion protein using the harvested mouse sera. As shown in Figure 3B,C, the GFP-VP1 protein reacted with both the anti-GFP antibody and the immunized mouse sera, indicating that the immunized mice could serve as a source for preparing monoclonal antibodies in the following steps.

### 3.3. Preparation and Characterization of Monoclonal Antibodies Against the VP1 Protein

**Figure 4:**
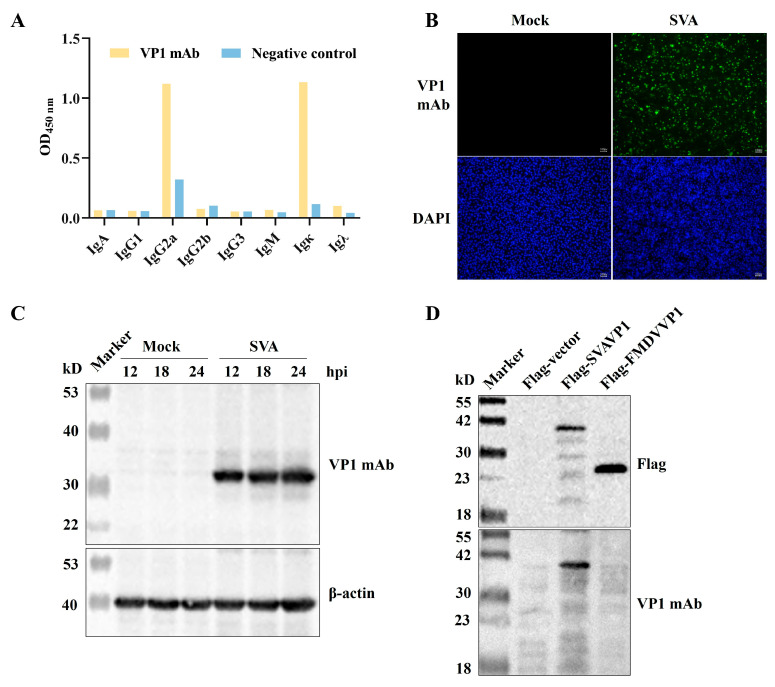
Characterization of a monoclonal antibody against the VP1 protein. (**A**) Isotype of the mAb determined with a mouse mAb isotyping ELISA kit. (**B**,**C**) Indirect immunofluorescence assay (IFA) (**B**), and Western blot (**C**) analyses of the specific reactivity of the mAb to the VP1 protein in the SVA-infected cells, in Figure B, green indicates VP1 mAb-positive signals, and blue labels cell nuclei. (**D**) Assessment of the specificity of mAb with VP1 proteins from SVA and FMDV.

The mice with relatively high antibody titers after the third booster were selected for a supplementary booster immunization. The splenocytes were aseptically collected and fused with SP2/0 cells. Positive hybridomas were selected using indirect ELISA and subsequently subcloned. After three consecutive cycles of screening and subcloning, a single hybridoma clone with stable VP1-specific mAb secretion was acquired. This clone was expanded in vitro and inoculated into mice for ascites preparation. Indirect ELISA determined the ascites antibody titer as 1:1,024,000, and isotype analysis confirmed the mAb belongs to IgG2a heavy chain paired with κ light chain (Figure 4A).

### 3.4. The Immunoreactivity of VP1 mAb with SVA

The immunoreactivity of the VP1 mAb with SVA was evaluated using the SVA strain CHhb17. For the immunofluorescence assay (IFA), BHK-21 cells were infected with CHhb17 and collected at 24 h post-infection. As shown in Figure 4B, specific green fluorescence signals were observed in SVA-infected cells incubated with the VP1 mAb. In contrast, no fluorescence was detected in uninfected cells, indicating that the VP1 mAb specifically recognizes SVA infection. To further characterize the binding properties of the VP1 mAb, Western blot analysis was conducted on lysates of virus-infected cells harvested at 12, 18, and 24 h post-infection, as well as lysates from uninfected cells. The findings revealed that the VP1 mAb specifically detected the virally expressed VP1 protein, and its expression level progressively increased with prolonged infection time; no reactivity was observed with proteins from uninfected cells, confirming that this antibody exhibits high specificity with the viral protein and can be used for the detection of viral infection (Figure 4C).

### 3.5. The Specificity of VP1 mAb

To assess the specificity of the mAb against SVA VP1, we analyzed its reactivity with the VP1 protein of FMDV, another vesicular disease-causing virus belonging to the same family, *Picornaviridae*. As shown in Figure 4D, the VP1 mAb specifically recognized SVA VP1 but did not react with FMDV VP1, indicating that the mAb exhibits good specificity.

### 3.6. Mapping of the VP1 mAb Epitope

**Figure 5:**
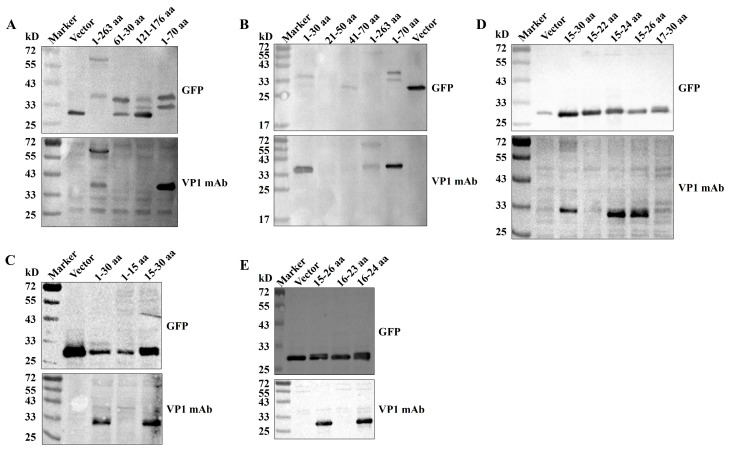
Precise epitope mapping of the mAb. (**A**) Three overlapping peptides, T1-1 (1–70 aa), T1-2 (61–130 aa), and T1-3 (121–176 aa), were expressed as GFP-fused proteins to assess their reactivity with the mAb using Western blot. (**B**) Three overlapping peptides covering T1-1 (1–70 aa) were used for the second round of mapping. (**C**) Two overlapping peptides that covered T2-1 (1–30 aa) were used for the third round. (**D**) Four overlapping peptides that covered T3-2 (15–30 aa) were further tested for fine epitope mapping. (**E**) Defining the shortest epitope region recognized by the mAb.

A protein truncation expression strategy was employed to localize the epitope targeted by the VP1 mAb. VP1 fragments of various lengths were expressed as GFP-tagged fusion proteins (Figure 1). Western blot analysis showed that the VP1 mAb did not bind to the empty vector, T1-2, or T1-3, but specifically bound to T1-1, indicating that the recognized epitope was located within amino acids 1–70 of the VP1 protein (Figure 5A). This 70-amino-acid region was further divided into three segments: T2-1 (1–30 aa), T2-2 (21–50 aa), and T2-3 (41–70 aa). Western blot analysis revealed that the VP1 mAb recognized amino acids 1–30 (Figure 5B). Subsequently, this 30-amino-acid region was further truncated into T3-1 (1–15 aa) and T3-2 (15–30 aa), and the VP1 mAb was found to recognize amino acids 15–30 (Figure 5C). To determine the minimal epitope, two amino acids were sequentially deleted from either end of the T3-2 peptide, and the resulting fragments were expressed and analyzed by Western blot. The findings demonstrated that the VP1 mAb exhibited specific binding to T4-2 (15–26 aa) and T4-3 (15–24 aa), but not to T4-1 (17–30 aa) and T4-4 (15–22 aa), indicating that the binding site of the VP1 mAb is located within amino acids 16–24 (Figure 5D). The fact that T4-1 (17–30 aa) or T4-4 (15–22 aa) did not contain the complete epitope sequence suggested that amino acids 16–24 or 16–23 constitute the minimal epitope recognized by VP1 mAb. Therefore, additional truncated peptides T5-1 (16–24 aa) and T5-2 (16–23 aa) were constructed (Figure 5E). Western blot results demonstrated that the minimal binding site of the VP1 mAb is ^16^DTDFSGELA^24^ (Figure 5E).

### 3.7. Conservation Analysis of the VP1 Epitope

**Figure 6:**
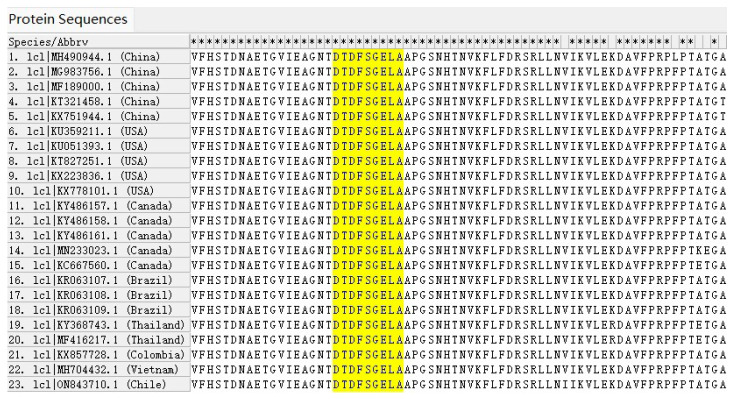
Sequence alignment of VP1 proteins from representative SVA isolates of different countries. The epitope region is highlighted in yellow, and relevant GenBank accession numbers and geographical sources are displayed on the left. Asterisks denote amino acid sites that are completely conserved across all displayed aligned sequences.

To analyze the conservation of the identified VP1 epitope, VP1 protein sequences available in GenBank were aligned using MEGA12. As shown in Figure 6, the epitope ^16^DTDFSGELA^24^ was present in representative strains from China, the United States, Canada, Brazil, Thailand, Colombia, Vietnam, and Chile. As of 31 December 2025, a total of 663 VP1 sequences (including the complete genomic sequences of SVA) were deposited in GenBank. Of these, 660 sequences contained this epitope, and only three sequences carried a single amino acid mutation (Table 2), indicating that the epitope identified here is highly conserved across SVA strains.

### 3.8. Spatial Distribution Analysis of the Newly Identified Epitope on the VP1 Protein

**Figure 7:**
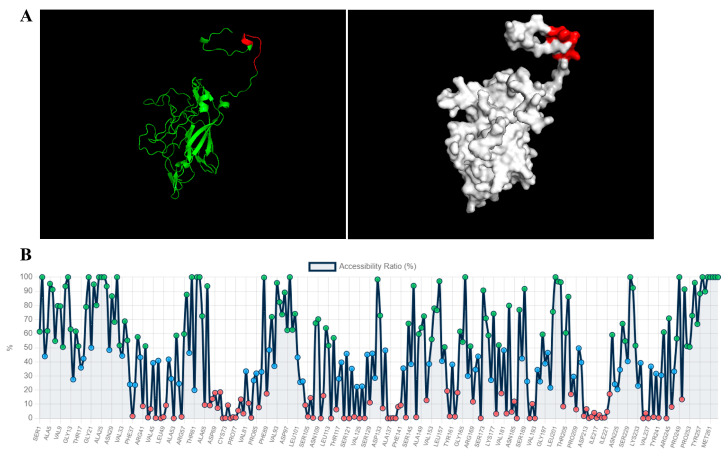
Spatial distribution analysis of the identified epitope on the VP1 protein. (**A**) Display of the epitope in the VP1 protein three-dimensional structure using PyMOL software. The epitope is colored red, while the other residues of VP1 are displayed in green (left panel) and white (right panel), respectively. (**B**) Analysis of the relative solvent accessibility (RSA) of each VP1 amino acid using the GETAREA program. Red circles denote amino acids with RSA values below 20%, green circles represent those with RSA ranging from 20% to 50%, and blue indicates residues with RSA above 50%.

To characterize the spatial distribution of the newly identified epitope ^16^DTDFSGELA^24^, the three-dimensional structure of the VP1 protein was predicted using AlphaFold according to its amino acid sequence. The epitope was subsequently mapped onto the VP1 structure using PyMOL software. As shown in Figure 7A, the epitope DTDFSGELA (depicted in red) is located in the α-helix and loop region of the protein. The GETAREA program analysis showed that the relative solvent accessibility (RSA) values of individual residues within this epitope were 51.1%, 35.9%, 42.3%, 78.9%, 100%, 50%, 94.9%, 80.1%, and 100%, respectively. As reported previously, residues with an RSA above 25% are considered surface-exposed [31]. Collectively, these results indicate that this epitope resides on the surface of VP1 (Figure 7B).

## 4. Discussion

The VP1 protein of SVA is present on the outer surface of the virion and serves as an essential component of the viral capsid [20]. Due to its critical role in capsid assembly, host cells deploy diverse mechanisms to restrict SVA infection via VP1 degradation [32]. For instance, SQSTM1 delivers SVA VP1 and VP3 to phagophores for degradation and thus suppresses viral replication [33]. Similarly, OPTN interacts with VP1 and promotes its degradation to inhibit viral replication [34]. Moreover, the Mx1 protein exerts anti-SVA activity by interacting with VP1 through its GTPase, oligomerization, and interaction domains [35]. Beyond capsid assembly, VP1 mediates viral entry by engaging anthrax toxin receptor 1 through its BC loop and loop II [22,23,36]. Furthermore, VP1 has been demonstrated to activate the AKT-AMPK pathway, inhibit mTOR, and induce autophagy [37]. Overall, VP1 executes multiple biological functions across the entire SVA life cycle. Further exploration of the biological functions of VP1 will advance our knowledge of SVA pathogenesis and immune mechanisms. This knowledge will, in turn, facilitate vaccine, therapeutic, and diagnostic development.

As an immunogenic structural protein of SVA, VP1 triggers both B- and T-cell immune responses, implying it harbors multiple B and T cell epitopes [19,38]. Several methods have been employed to identify antigenic epitopes of the VP1 protein, and all reported epitopes are summarized in Supplementary Table S1. For example, the linear epitope GELAAP, which resides on the surface of VP1 with a high antigenic index, was identified using a monoclonal antibody [39]. Another study combined epitope prediction with overlapping synthetic peptides and successfully characterized four major B-cell epitopes spanning residues 7–26, 48–74, 92–109, and 129–144, respectively [40]. In the present study, we expressed a series of truncated VP1 proteins in eukaryotic cells and identified the minimal epitope recognized by the VP1 mAb as ^16^DTDFSGELA^24^ by Western blot. Currently, the Immune Epitope Database http://www.iedb.org/ (accessed on 2 April 2026) contains 52 VP1 antigenic epitopes derived from five SVA strains. These epitopes are reactive with porcine sera, polyclonal antibodies, and monoclonal antibodies, and were primarily identified using peptide microarray, ELISA, Western blot, and phage display [41]. Several previously reported epitopes fully cover the motif identified herein: TGVIEAGNTDTDFSGELAAP and VIEAGNTDTDFSGELA from strain CH-FuJ/SVA/2017 (GenBank No. MH747510) [40], the epitope DTDFSGELAAPGSNHTNVKF from strain SVA-001 (GenBank No. DQ641257) [42], and the epitope EAGNTDTDFSGELAAPGSNH from strain SVA/HLJ/CHA/2016 (GenBank No. KY419132) [43]. Compared with earlier reported regions, this newly identified epitope is shorter and more precise. It facilitates the elucidation of the molecular mechanism of antibody recognition, eliminates interference from redundant sequences, and enables accurate chemical synthesis and modification of target peptides. To assess the conservation of this epitope, we analyzed VP1 protein sequences in GenBank using MEGA12. The results showed that 99.55% of the sequences carry the complete ^16^DTDFSGELA^24^ sequence, while only three sequences harbor a single amino acid mutation, demonstrating that the epitope is well preserved across different SVA isolates. AlphaFold structural prediction, together with PyMOL and GETAREA analyses, demonstrates that the epitope resides in the α-helix and loop regions of VP1 and is surface-exposed, which facilitates recognition by the host immune system.

Epitope-based vaccines are considered a promising strategy against viral infections, and several candidates targeting SVA have been reported. For instance, the identified epitope fragments VP1 amino acids 7–26, 48–74, and 92–109 were linked in tandem to construct a multi-epitope recombinant vaccine, which induced strong neutralizing antibody responses and provided effective protection against homologous SVA challenge [40]. Another study constructed an SVA nanoparticle vaccine by coupling a VP1 epitope (residues 21–26) and VP2 protein to the β-annulus peptide of tomato bushy stunt virus [44]. Following two immunizations, high titers of SVA-specific neutralizing and IgG antibodies were elicited in both mice and swine. A protection experiment in swine revealed that the nanoparticle vaccine and an inactivated SVA vaccine both conferred 80% protection against SVA challenge. Clearly, epitope mapping represents the first step in epitope-based vaccine development [45]. Nevertheless, the vaccine potential of the epitope identified in our study remains to be verified via virus neutralization assays and animal protection experiments. At present, its main advantage is high conservation, whereas its limitation lies in being a linear epitope, which is unlikely to be neutralizing. This constitutes a limitation of our study.

## 5. Conclusions

In summary, we developed a mAb targeting the SVA VP1 protein and identified its linear B-cell epitope ^16^DTDFSGELA^24^. This antibody and the identified epitope collectively offer a valuable tool for etiological studies of SVA and functional research on its VP1 protein.

## Figures and Tables

**Table 1:** Primers used in this study.

Primers	Sequences (5′-3′)
pET28a-VP1-F	cagcaaatgggtcgcggatccACCGACAACGCCGAGACTG
pET28a-VP1-R	ctcgagtgcggccgcaagcttTTGCATCAGCATCTTTTGCTTG
T1-1(1–70)F	ctaccggactcagatctcgagATGTCCACCGACAACGCCG
T1-1(1–70)R	ggatcccgggcccgcggtaccGACCATCGTCCTGCTGTGCA
T1-2(61–130)F	ctaccggactcagatctcgagATGACAGCAACAGGTGCACAGC
T1-2(61–130)R	ggatcccgggcccgcggtaccGCAGTGAGACCACCGTGACTTCA
T1-3(121–176)F	ctaccggactcagatctcgagATGTCAGACCTTGAAGTCACGGT
T1-3(121–176)R	ggatcccgggcccgcggtaccGTCCACCCTTGCTGGTGAAAG
T2-1(1–30)R	ggatcccgggcccgcggtaccGATGGTTAGAGCCAGGAGCCG
T2-2(21–50)F	ctaccggactcagatctcgagATGGGTGAACTGGCGGCT
T2-2(21–50)R	ggatcccgggcccgcggtaccGCTCCAGTACCTTAATTACATTCAGTAGT
T2-3(41–70)F	ctaccggactcagatctcgagATGCGACTACTGAATGTAATTAAGGTACT
T3-1(1–15)F	tcgagTCCACCGACAACGCCGAGACTGGTGTTATTGAGGCAGGTAACACTcggtac
T3-1(1–15)R	cgAGTGTTACCTGCCTCAATAACACCAGTCTCGGCGTTGTCGGTGGAc
T3-2(15–30)F	tcgagACTGACACCGATTTCTCTGGTGAACTGGCGGCTCCTGGCTCTAACCATcggtac
T4-1(17–30)F	tcgagACCGATTTCTCTGGTGAACTGGCGGCTCCTGGCTCTAACCATcggtac
T4-1(17–30)R	cgATGGTTAGAGCCAGGAGCCGCCAGTTCACCAGAGAAATCGGTc
T4-2(15–26)F	tcgagGACACCGATTTCTCTGGTGAACTGGCGGCTCCTcggtac
T4-2(15–26)R	cgAGGAGCCGCCAGTTCACCAGAGAAATCGGTGTCc
T4-3(15–24)F	tcgagACTGACACCGATTTCTCTGGTGAACTGGCGcggtac
T4-3(15–24)R	cgCGCCAGTTCACCAGAGAAATCGGTGTCAGTc
T4-4(15–22)F	tcgagACTGACACCGATTTCTCTGGTGAAcggtac
T4-4(15–22)R	cgTTCACCAGAGAAATCGGTGTCAGTc
T5-1(16–24)F	tcgagGACACCGATTTCTCTGGTGAACTGGCGcggtac
T5-1(16–24)R	cgCGCCAGTTCACCAGAGAAATCGGTGTCc
T5-2(16–23)F	tcgagGACACCGATTTCTCTGGTGAACTGcggtac
T5-2(16–23)R	cgCAGTTCACCAGAGAAATCGGTGTCc

The homology arm sequences or restriction endonuclease sites are lowercased.

**Table 2:** Alignment of the epitope region in VP1 sequences from the GenBank database.

Variations	Sequences	Amino Acid Substitutions								
No substitution	660	D	T	D	F	S	G	E	L	A
Single substitution	1 (MK562465)	-	-	-	-	-	-	K	-	-
	1 (MH634530)	-	-	-	-	-	-	G	-	-
	1 (MK487483)	-	-	-	-	Y	-	-	-	-
Total	663									

## Data Availability

The data that support the findings of this study are available from the corresponding author upon reasonable request. The monoclonal antibody against Senecavirus A VP1 is available from the corresponding author upon reasonable request.

## Supplementary Materials

The following supporting information can be downloaded at: https://www.mdpi.com/article/10.3390/ani16121856/s1, Table S1: Summary of identified epitopes on SVA VP1 [46].
