# Supplementary material for: Identification of a Conserved Linear Antigenic Determinant in the Senecavirus A VP1 Protein
Source: Animals (Basel). 2026 Jun 16;16(12):1856. doi: 10.3390/ani16121856 (PMC13296050; doi:10.3390/ani16121856)
Supplement: Supplementary file 1 [file animals-16-01856-s001.zip › animals-4354235-supplementary.pdf]

Table S1 Summary of identified epitopes on SVA VP1

Sequences	Location	Identification methods	Isolates (GenBank No.)	References
GELAAP	21-26	Western blot	SVV−CH-SD (MH779611)	[39]
FGLYVNPSDSGVLANT	89-104			
RLLNVIKVLEKDAVFP	41-56			
SEYQASSFVYDQLHVP	145-160			
SLEPDLEFAVGWFPSG	129-144			
STDNAETGVIEAGNTD	1-16	ELISA		
TDFSGELAAPGSNHTN	17-32			
VASRPATRFGLYVNPS	81-96		CH-FuJ/SVA/2017 (MH747510)	[40]
VIEAGNTDTDFSGELA	9-24			
LEKDAVFPRPLPTATG	49-64			
TGVIEAGNTDTDFSGELAAP	7-26			
		Western blot		
YVNPSDSGVLANTSLDFN	92-109			
VLEKDAVFPRPLPTATGAQQDDGYFCL	48-74			
DTDFSGELAAPGSNHTNVKF	16-35			
FAVGWFPSGSEYQASSFVYD	136-155			
GGASKLSSATRGLPAHADWG	196-215			
STDNAETGVIEAGNTDTDFS	1-20	ELISA		
			SVV-001 (DQ641257)	[42]
SYKQKMLMQ	256-264			
IKVLEKDAVFPRPFPTQEGA	46-65			
LYANPSGSGVLANTSLDFNF	91-110			
RTPRAFASKGGKVSFVLPWN	166-185			

SFVYDQLHVPFHFTGRTPRA	151-170	ELISA and Microarray	SVV-001 (DQ641257); SVA/HLJ/CHA/2016 (KY419132)	[40,43]
TPRPTVASRPATRFGLYANP	76-95			
TQEGAQQDDGYFCLLTPRPT	61-80			
LDFNFYSLACFTYFRSDLEV	106-125			
SDLEVTVVSLEPDLEFAVGW	121-140			
TNVKFLFDRSRLLNVIKVLE	31-50			
VLPWNSVSSVLPVRWGGASK	181-200			
YKNARAWCPSMLPFRSYKQK	241-260			
WNSVSSVLPVRWGGASKL	184-201	Phage display	-	[46]
HFTGRTPRAFAS	162-173			
AVKHVAVYVRYKNARAWCPS	231-250	Microarray	SVA/HLJ/CHA/2016 (KY419132)	[43]
EAGNTDTDFSGELAAPGSNH	11-30			
EPDLEFAVGWFPSGSEYQAS	131-150			
FPSGSEYQASSFVYDQLHVP	141-160			
FTSKGGKVSFVLPWNSVSSV	171-190			
GELAAPGSNHTNVKFLFDRS	21-40			
HADWGTIYAFIPRPNEKKST	211-230			
IPRPNEKKSTAVKHVAVYVR	221-240			
KDAVFPRPFPTATGAQQDDG	51-70			
LANTSLDFNFYSLACFTYFR	101-120			
LPVRWGGASKLSSATRGLPA	191-210			
LSSATRGLPAHADWGTIYAF	201-220			
LYVNPSDSGVLANTSLDFNF	91-110			
MLPFRSYKQKMLMQ	251-264			
RLLNVIKVLEKDAVFPRPFP	41-60			
SFVYDQLHVPYHFTGRTPRA	151-170			

TATGAQQDDGYFCLLTPRPT	61-80			
VASRPATRFGLYVNPSDSGV	81-100			
YFCLLTPRPTVASRPATRFG	71-90			
YHFTGRTPRAFTSKGGKVSF	161-180			
YSLACFTYFRSDLEVTVVSL	111-130			
DTDFSGELA	16-24	Western blot	CHhb17 (MG983756.1)	This study
